# A Comparative Performance Analysis of Load Cell and Hall-Effect Brake Sensors in Sim Racing

**DOI:** 10.3390/s25133872

**Published:** 2025-06-21

**Authors:** John M. Joyce, Adam J. Toth, Mark J. Campbell

**Affiliations:** 1 Lero the Research Ireland Centre for Software, University of Limerick, V94T9PX Limerick, Ireland; john.m.joyce@ul.ie (J.M.J.); mark.campbell@ul.ie (M.J.C.); 2 Department of Physical Education & Sport Sciences, University of Limerick, V94T9PX Limerick, Ireland; 3 Centre for Sport Leadership, Stellenbosch University, Matieland 7602, South Africa

**Keywords:** esports, video gaming, car racing, brakes, Hall sensor

## Abstract

Alongside the general growth in gaming and esports, competitive simulated (sim) racing has specifically surged in popularity in recent years, leading to an increased demand for understanding performance. In recent work, braking-related metrics were identified among the key indicators of successful sim racing performance. While load cell sensors currently serve as the industry standard for brake hardware, sensors like the Hall sensor may provide another viable option. No study to date has compared the performance of these braking sensors. The aim of this study was to investigate whether sim racing performance differed when racing using a load cell or Hall brake sensor. Twenty (*N* = 20) experienced sim racers raced with both the load cell and Hall brake sensors (with load cell behaviour mimicked on the Hall sensor) in a repeated measures design. Paired samples *t*-tests, Wilcoxon-signed rank tests, and chi-square goodness-of-fit tests were used to test for differences in lap time, driving behaviour metrics, and subjective responses between the two sensors. Results showed that participants achieved faster lap times using the load cell brake sensor (average lap time (*p* = 0.071); fastest lap time (*p* = 0.052)) and displayed braking behaviour more aligned with that of a “faster racer”. The differences observed may be potentially attributed to differences in in-game response curves between two brake sensors, which specifically may impact both the initial, and trail braking, phases.

## 1. Introduction

The objective of simulated (sim) racing is to mimic the experiences produced by real-world racing in a virtual environment. The similarities between real-world and sim are evidenced by the participation of F1 drivers, such as Max Verstappen and Lando Norris, in sim racing events, and their use of simulated racing equipment to hone their skills when unable to race on a physical racing track [1] (pp. 156–157). Moreover, professional sim racing teams now have engineers working alongside them to optimise every facet of performance [2].

Sim racing has surged in popularity in recent years, which can be seen by its expanding user base and increase in competition event viewership [3]. By way of example, the all-time peak player count for the F1 series of games has increased from 8085 in 2017 to 23,763 in 2022 [4]. iRacing also boasts an estimated 133,000 monthly players on their online service as of December 2023 [5]. This popularity and worldwide attention has led to an increased appetite to better understand the elements of sim racing performance, specifically those related to the racer and equipment (both hardware and software).

Although a plethora of research exists in simulated driving [6,7,8], simulated racing differs in that it is competitive [9] and racers must operate the virtual vehicle to navigate tracks at the highest attainable speeds [10]. Research in sim racing performance is in its infancy, with few studies published to date (e.g., [11,12,13,14]). Among the early research is the seminal work by Hojaji and colleagues [15], which leveraged data analytics and machine learning techniques to uncover the most influential in-game telemetric parameters for sim racing performance across different levels of racer expertise. This identification of key performance metrics in sim racing is among early work using artificial intelligence to understand how to optimise performance in gaming and esports [16]. As the steering wheel and pedal set represent the two inputs through which racers interact with the game, it is logical that key performance indicators relating to steering and braking behaviour were found to be among the most influential to performance. For braking, Remonda and colleagues [17] concluded that braking is the most impactful skill to improve the quality of lap times in their study of sim racing using machine learning, given that both their AI model and human participants took many more laps to learn how to brake efficiently. In the study by Hojaji and colleagues, faster drivers were specifically found to apply greater brake amplitudes across braking events over a lap and exhibited longer trail braking durations (a technique used to better rotate the vehicle in slower speed corners [18]) in corner sections [15]. Additional important braking indicators include the time taken to reach peak brake amplitude and total braking duration [19]. Given the importance of braking related behaviours in simulated racing, it is also important to better understand how brake pedal sensor design in simulated racing setups might influence performance.

There are many brakes available on the sim racing market today, each differing according to the type of sensors they use to convert the mechanical input from the foot to an analogue or digital signal [20] in the game to decelerate the virtual car. Many entry level pedal systems use potentiometers, but, recently, Hall-effect sensors (referred to as “Hall sensor” hereafter) have become more widely explored as they are a non-contact sensor with increased durability (Supplementary Figure S1a,b). Hall sensors work by applying a magnetic field perpendicularly to a conductive material that carries a current from one of its ends to another (see Supplementary Figure S2). The current produces a force called the Lorentz force [21], which can be converted to, and is measured as, a voltage. In a sim racing Hall sensor brake pedal, a magnet is placed on the pedal shaft (Supplementary Figure S1a), and when the brake pedal is pressed, the magnet moves relative to the fixed Hall sensor at the bottom of the shaft (Supplementary Figure S1b). The magnet’s movement creates variations in the strength of the magnetic field that the Hall sensor detects. The stronger the magnetic field (i.e., the more the pedal has travelled and closer the magnet is to the Hall sensor), the greater the amplitude of braking applied. This technology is being used in electronic accelerator pedals in road cars [22].

One proposed limitation of Hall sensors is that they solely measure the angular distance that the brake travels [21], whereas, in real-world vehicles, electro-hydraulic brake systems detect the amount of force applied to the brake [23]. The need for more “realistic” braking mechanisms in sim racing has led to the implementation of force-based sensors. The sensor that is currently used in most mid-range braking systems is the load cell sensor (Supplementary Figure S3). The load cell consists of a strain gauge that is connected to an elastic spring element comprising aluminium or steel [24]. As force or strain is applied, the spring element deforms (Poisson effect), which alters the resistance of the strain gauge. In the context of the sim racing brake pedal, as force is applied to the brake, the spring element that is placed at the bottom of the brake shaft deforms (Supplementary Figure S3), registering a change in voltage that can be measured and calibrated in-game to decelerate the virtual car. The behaviour of the load cell brake more closely mimics real-world braking and is thought to create a more realistic and immersive experience for the sim racer [25], although this is yet to be proven. Moreover, load cell brakes are more costly to manufacture (e.g., 100 kg load cells range from GBP 153 to GBP 1040 [26] compared with prices ranging from GBP 0.42 to GBP 18.64 for Hall sensors [27]) and less durable compared with Hall sensors. To date, no research has examined whether sim racing performance is superior when using either of these brake sensors.

The purpose of this study is to compare the sim racing performance and braking behaviour characteristics of participants when using load cell and Hall sensor brakes, under conditions where the Hall sensor is calibrated to mimic load cell behaviour. Firstly, we hypothesise that overall performance will differ when racing using the two brake sensors. This will be evidenced by a difference in average and fastest lap times between brake sensors. Secondly, we hypothesise that the consistency of lap time performance will differ between the two brake sensors. This will be evidenced by a difference in the lap time coefficient of variation (CV) between brake mechanisms. CV is a widely used metric to quantify within-subject variation in behavioural and sports science studies [28,29]. Thirdly, we hypothesise that braking behaviour will differ between the two sensors. This will be evidenced by observing a difference in average brake amplitudes (BAs), trail braking durations (TBDs), time to peak brake (TPB), and overall braking durations (BDs) between brake sensors. Fourthly, we hypothesise that the consistency of these measures will differ between the two brake sensors. This will be evidenced by a difference in CVs between brake sensors for BA, TBD, TPB, and BD. Lastly, we hypothesise that participants will be unable to distinguish between the brake sensors based on how they feel under foot and respond in-game, and be unable to correctly distinguish between the load cell and Hall sensor brakes following the conclusion of testing.

## 2. Materials and Methods

### 2.1. Participants

Twenty (N = 20) participants (age 21.45 ± 3.61; mean ± SD) with prior sim racing experience were recruited from a university population and the surrounding region. Participants had 3.89 ± 1.82 years of sim racing experience and played sim racing games for 6.20 ± 5.89 h per week. Participants reported having no neuropsychological and neuromuscular disorders and provided informed written consent prior to participation. Approval for this study was authorised by the research ethics board (2024_12_13_EHS) at the University of Limerick in accordance with the Declaration of Helsinki.

### 2.2. Materials

**Figure 1:**
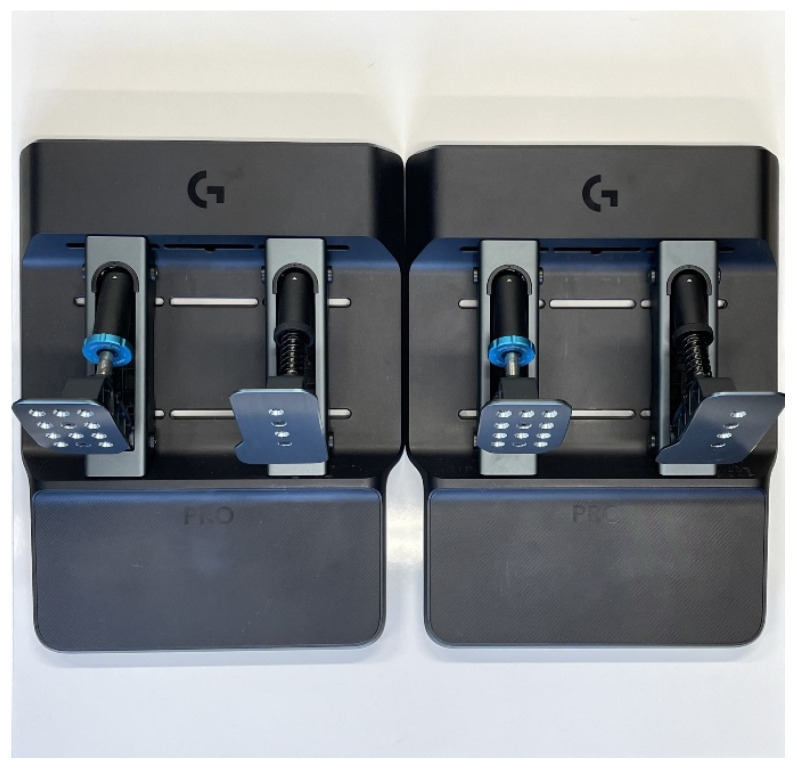
Image of pedal bases with load cell brake (**left**) and Hall sensor brake (**right**). Both pedal bases used in this study appeared visually identical to participants.

Participants raced on the Brands Hatch circuit with the McLaren 720 s GT3 car using the racing game Assetto Corsa Competizione (Kunos Simulazioni; Rome, Italy) (v1.9.6). The game was run at 120 Hz on a 32-inch monitor (MSI; New Taipei City, Taiwan). Participants sat in a sim racing rig (Sim Race Components; Milan, Italy) with a Logitech G Pro wheelbase (Logitech; Lausanne, Switzerland). Two Logitech G Pro pedal bases, identical in appearance (Figure 1), were used in this study. Each had a throttle and brake, with the default load cell brake on one and the clutch pedal (which relies on a Hall sensor) on the other. Due to the sensors used in the Logitech pedals being proprietary in nature, no sensor specifications were readily available, therefore we used the DXTweak2 program [30] to calculate the resolutions of each sensor. The load cell sensor utilises a strain gauge and uses a 12-bit rate (4096 values) for gaming. The maximum load the sensor can withstand is 100 kg, which equates to 24 g per bit. The Hall sensor is also 12-bit (4096 values), with 25 degrees of rotation equating to 0.006 degrees per bit. 

**Figure 2:**
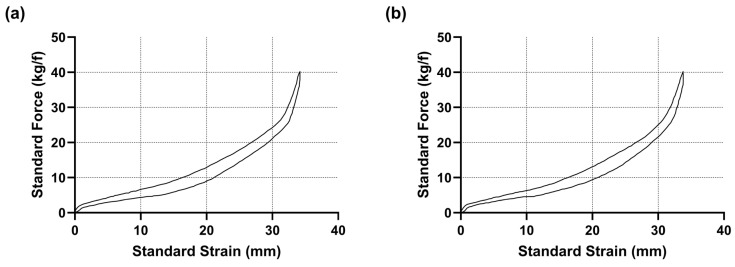
(**a**) Force–travel curve for the load cell brake. (**b**) Force–travel curve for the Hall sensor brake. As can be seen, curves for both brakes look identical, allowing both brakes to feel the same under braking.

To ensure that both the load cell and Hall sensor brakes were mechanically identical, we adapted the clutch pedal on the second pedal base. The spring mechanism from the clutch pedal was removed and replaced with a load cell piston (see Supplementary Figure S4). This also ensured that the pedal sets were visually identical. Within the load cell piston, elastomers were placed to alter the amount of travel the pedal experiences when subjected to a given force. The same combination of elastomers (allowing 24 mm travel as marketed by Logitech) was placed in both the load cell and Hall sensor pistons, and a uniaxial force–displacement machine (ZwickRoell Ltd.; Worcester, UK) was used to depress the brake pedals at a constant velocity until the brakes were fully depressed. The resultant loads were measured with Grade1/0.5 ISO 7500-1 [31] accuracy load cells. The load cells were set to zero prior to contact with the pedals, regardless of approach displacement, and force was measured from the initial pedal face contact. During this process, the force–travel curve for each brake sensor was recorded to verify their similarity (Figure 2). 

To mimic load cell brake behaviour on the Hall sensor, we altered the way in which the Hall sensor brake converted the physical input from the racer to in-game brake amplitudes using a custom Python program. This Python script converted force values for the load cell brake to travel values for the Hall sensor brake based on the corresponding brake force and elastomer combination (corresponding to 38 kg of braking force and 24 mm travel elastomers). For example, to achieve 20 kg of brake force with this elastomer combination, 19.3 mm of travel in the brake is required. Therefore, 19.3 mm of travel on the Hall sensor brake would correspond to 20 kg of brake force. 

Both the load cell and Hall sensor brakes were calibrated using Logitech’s “G-HUB” software (v.2024.3.553733) [32]. This allowed us to set the load cell sensor to 38 kg of force (i.e., 38 kg of pressure is needed to reach maximum braking. Following the conversion of force values to travel values on the Hall sensor, we used this software to check whether the Hall sensor was responding in-line with the load cell brake. The brakes were also calibrated in-game to ensure that they were reaching maximum braking response prior to each participants’ data collection.

Telemetry data were captured using MoTeC i2 Pro software at a sample rate of 50 Hz (MoTeC; Croydon South, Australia) (v.1.1.5.0085), from which performance and braking metrics could be calculated. A shared-memory program was run in parallel with MoTeC i2 Pro software, which facilitated the detection and exclusion of invalid laps (where all four wheels of the car have exceeded track limits).

### 2.3. Protocol

After providing their informed consent, participants filled out a demographics questionnaire that gathered information about participants’ sim racing experience and sim racing setups. Participants were then randomly assigned a brake sensor order and completed 14 laps around the Brands Hatch circuit in a McLaren 720 s GT3, where they were instructed to produce fast but clean (keeping the car on track) lap times with the first brake sensor condition. Participants used the first 7 laps to familiarise themselves with the track and car, while telemetry data from the last 7 laps were recorded for analysis. After completing 14 laps using the first brake sensor, each participant was asked to briefly leave the lab. During this time, the experimenter switched out the pedal bases on the sim racing rig. For the second brake condition, participants drove 7 laps around the track. Ten participants raced using the load cell brake first and the Hall sensor second, while another ten were exposed to the opposite order. Participants were asked questions related to their perception of the two conditions both during and after the completion of the conditions (see Table 1). Question 1 was asked following the completion of each condition, while questions 2–5 were asked at the end of the session following the completion of both conditions. Questionnaire responses can be found in Supplementary File S1. A graphical representation of the protocol can be seen in Supplementary Figure S5.

### 2.4. Data Processing

Invalid laps and lap times that exceeded two standard deviations of the average lap time for a condition were excluded from further analyses. One participant’s data were completely excluded due to the fact they did not complete a valid lap in either condition. Data from 19 participants were carried forward for performance and braking behaviour analysis. Data from 14 participants were brought forward for consistency analyses as they achieved 3 or more valid laps in each condition. The average and fastest lap times were obtained from telemetry data for the load cell and Hall sensor brake conditions. Concerning the average lap time variable, each valid lap time for a participants was summed and averaged within a condition to obtain an accurate representation of a participant’s performance within that condition. Numerous braking behaviour metrics were calculated for each participant from the telemetry braking data. These calculations can be found in Table 2. 

**Figure 3:**
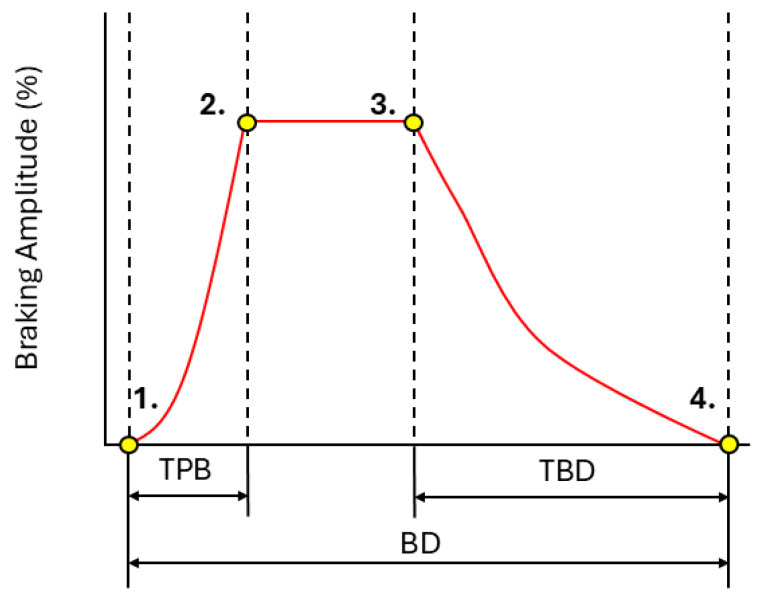
An example of a typical brake trace seen from telemetry data with the metrics of time to peak brake (TPB), trail braking duration (TBD), and braking duration (BD) highlighted, with specific data points outlined on the trace. Brake start (**1**), peak brake start (**2**), peak brake end (**3**), and brake end (**4**).

First, a braking event was defined as a window of non-zero braking amplitudes (%) that exceeded a peak amplitude of 10% with at least 100 frames of no braking amplitude either side. For each brake event, we defined the average brake amplitude (BA: average of all non-zero braking amplitudes), trail braking duration (TBD: the time taken to return to 0% braking amplitude from the last frame of the peak brake amplitude), time to peak brake (TPB: the time taken to reach maximum braking amplitude), and total brake duration (BD: total duration that the brake is applied). A visualisation of some of these metrics can be found in Figure 3. Each of these metrics was averaged over all brake events within a lap before being subjected to analysis.

### 2.5. Data Analysis

SPSS v.28.0 (SPSS, Chicago, IL, USA, 2021) was used to perform statistical analyses. A Shapiro–Wilk test was carried out alongside the investigation of Q-Q and stem-and-leaf plots to check the normality of the data.

To investigate whether there was an effect of brake sensor condition on the overall performance of participants, we compared the fastest and average lap times by participants for each brake using paired samples *t*-tests. 

To investigate whether the brake sensor condition influenced the performance consistency, a Wilcoxon signed-rank test was conducted on within-participants lap time CVs (coefficient of variations).

To investigate whether the brake sensor condition affected braking behaviour, paired samples *t*-tests were used to compare BA, TBD, TPB, and BD across brake sensor conditions.

To test whether there was an effect of brake sensor condition on the consistency of braking behaviour, we compared BA, TBD, TPB, and BD within-participant CVs across brake conditions using a Wilcoxon signed-rank test (lap time CV; BA CV) and paired samples *t*-tests (TBD CV; TPB CV; BD CV).

Finally, to investigate whether participant responses to perceptual questions differed between brake conditions, three chi-square goodness-of-fit tests were carried out on responses to questions 2, 3, and 5, where observed and expected responses were compared (Table 1).

Statistical significance was determined using an alpha level of *p* ≤ 0.05 with Sidak alpha level corrections for multiple comparisons. Cohen’s *d* effect sizes were reported, and results were expressed as means ± SD in the case of paired samples *t*-tests and as medians ± interquartile range in the case of Wilcoxon signed-rank tests.

## 3. Results

### 3.1. Overall Performance

**Figure 4:**
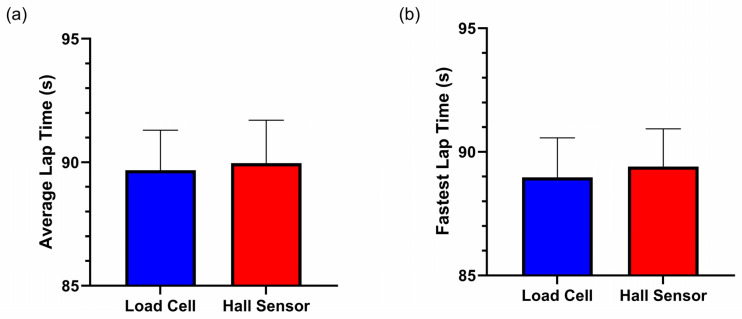
(**a**) Average lap times and (**b**) fastest lap times for the load cell and Hall sensor brakes. Error bars represent standard deviations.

Participants produced faster average lap times (*t*(16) = −1.932, *p* < 0.071, *d* < 0.469) (Figure 4a) and faster fastest lap times (*t*(16) = −2.099, *p* < 0.052, *d* = 0.509) (Figure 4b) when racing using the load cell brake compared with the Hall sensor brake (Table 3). 

### 3.2. Performance Consistency

**Figure 5:**
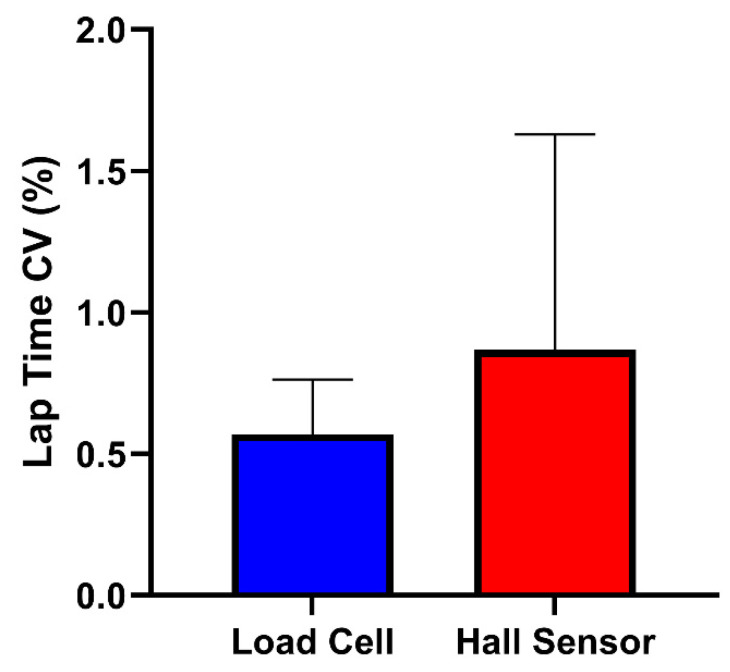
Within-participant CVs (coefficient of variations) for lap time across load cell and Hall sensor brakes. Error bars represent interquartile ranges.

No significant difference was found between the load cell and Hall sensor brakes when comparing lap time CVs (Z = −0.105, *p* = 0.917) (Table 4) (Figure 5).

### 3.3. Braking Behaviour

**Figure 6:**
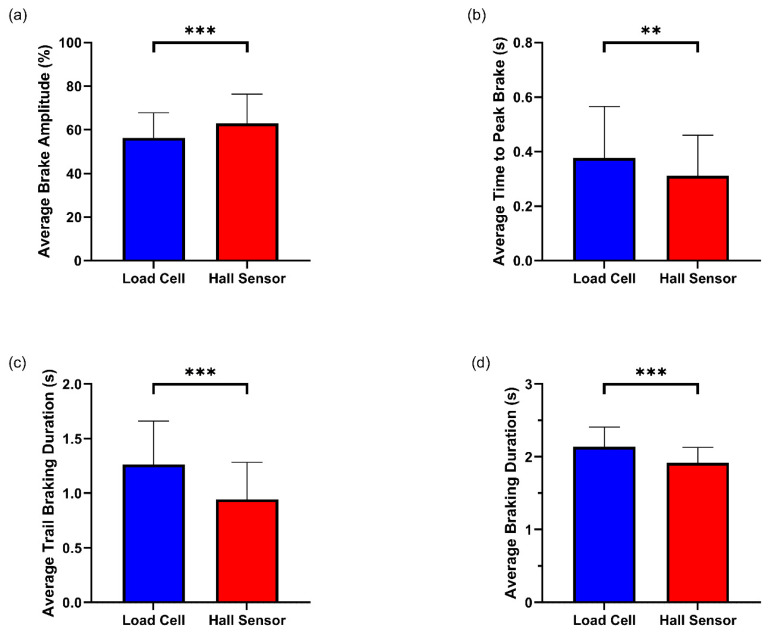
(**a**) Average BA, (**b**) average TPB, (**c**) average TBD, and (**d**) average BD across load cell and Hall sensor brakes. Error bars represent standard deviations. *** denotes a significant difference at *p* < 0.001, ** denotes a significant difference at *p* < 0.01.

Participants produced significantly higher average BA with the Hall sensor brake (*M* = 62.99, *SD* = 13.36) compared with the load cell brake (*M* = 56.27, *SD* = 11.50) (*t*(16) = −4.957, *p* < 0.001, *d* = 1.202) (Table 3) (Figure 6a). Participants reached peak brake amplitudes significantly faster with the Hall sensor brake (*M* = 0.31, *SD* = 0.15) compared with the load cell brake (*M* = 0.38, *SD* = 0.19) (*t*(16) = −3.396, *p* = 0.004, *d* = 0.824) (Table 3) (Figure 6b). Participants also displayed significantly longer TBDs with the load cell brake (*M* = 1.26, *SD* = 0.40) compared with the Hall sensor brake (*M* = 0.94, *SD* = 0.34) (*t*(16) = 9.891, *p* < 0.001, *d* = 2.399) (Table 3) (Figure 6c). Lastly, participants achieved significantly longer BDs with the load cell brake (*M* = 2.16, *SD* = 0.29) compared with the Hall sensor brake (*M* = 1.94, *SD* = 0.22) (*t*(14) = 6.164, *p* < 0.001, *d* = 1.592) (Table 3) (Figure 6d).

### 3.4. Consistency of Braking

**Figure 7:**
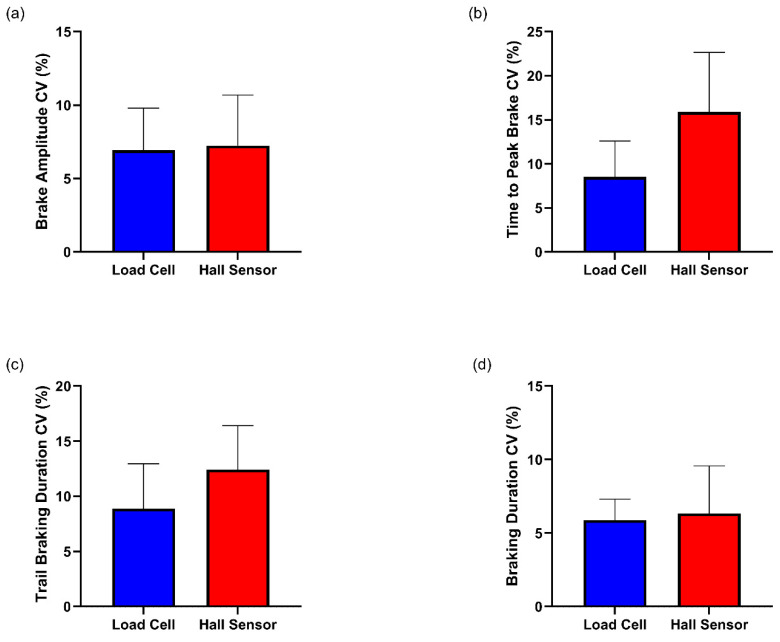
(**a**) Within-participant CVs for BA, (**b**) TPB, (**c**) TBD, and (**d**) BD across load cell and Hall sensor brakes. Error bars represent standard deviations.

BA CVs did not differ between the load cell and Hall sensor brake conditions (*Z* = −0.314, *p* = 0.753) (Table 4) (Figure 7a), neither did TPB CVs (*t*(5) = −1.794, *p* = 0.133, *d* = 0.732) (Table 3) (Figure 7b), TBD CV’s (*t*(5) = −1.426, *p* = 0.213, *d* = 0.582) (Table 3) (Figure 7c) or BD CVs (*t*(5) = −0.286, *p* = 0.786, *d* = 0.117) (Table 3) (Figure 7d). 

### 3.5. Perception of Brake Feel, In-Game Response, and Load Cell Identification

There was no significant difference in the number of participants who perceived that the two brakes felt different with those who did not (*X*^2^(1) = 2.882, *p* = 0.090) (Table 5). No significant difference was found in the number of participants who perceived a difference in how the two brakes responded in-game with those who did not (*X*^2^(1) = 0.059, *p* = 0.808) (Table 5). Lastly, no significant difference was found in the number of participants who correctly identified the load cell brake and the number of participants that incorrectly identified the load cell brake (*X*^2^ = 0.529, *p* = 0.467) (Table 5).

## 4. Discussion

**Figure 8:**
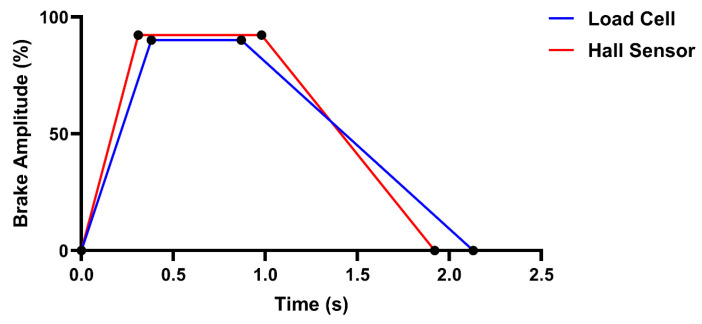
Comparison of the average brake traces for the load cell (blue) and Hall sensor (red) brakes.

In this study we sought to investigate whether sim racing performance and sim racer braking behaviour differed when using a load cell or Hall sensor brake. Our study’s findings provide partial support that the load cell brake may confer a performance advantage for racing, a view held by many in the sim racing community. When utilising the load cell brake, participants achieved faster lap times (Figure 4). When participants utilised the load cell brake, they achieved significantly lower average brake amplitudes (Figure 6a), reached peak braking significantly slower (Figure 6b), maintained significantly longer trail braking durations (Figure 6c) and, in turn, significantly longer total braking durations (Figure 6d) when compared with their utilisation of the Hall sensor brake. Figure 8 summarises these findings visually as two brake events with the different brake sensors. Taken together, the braking behaviours displayed when using the load cell brake align with the braking behaviour of a “faster driver”. Finally, although participants’ performance and braking behaviours significantly differed between the brake sensors, participants could not subjectively perceive a difference between the brake sensors. These findings are discussed in more detail in the sections below.

As highlighted above, the braking behaviours participants displayed when racing using the load cell brake arguably explain the faster lap times that participants were able to achieve with the load cell brake compared with the Hall sensor brake, as previous research has found that braking-related metrics contribute greatly to overall performance [12,15,17]. Although the Hall sensor matches “faster” braking performance in terms of the time to reach peak brake amplitude, it lagged behind in all other key braking metrics identified in Hojaji and colleagues [15]. Moreover, we found racers were no less consistent when racing with either brake sensor. Performance and braking metric CVs were low for both brake sensors, indicative of consistent performance [33]. 

Based on our results, the increased trail braking duration may be particularly important. Longer trail braking durations would result in more frames of low brake amplitude data contributing to average brake amplitudes within brake events, which would lower overall lap BAs for the load cell brake. Longer TBDs would also arguably lengthen BDs, where we also see a significant difference between the load cell and the Hall sensor. The trail braking technique is highly important for vehicle stability control [34]. When using the trail braking technique effectively, braking force is slowly reduced and modulated from maximal force to control the balance of the car as drivers increase the steering angle as they near the apex of a corner [35]. Reducing trail braking (i.e., applying the brakes rapidly and releasing them too quickly) will result in the car becoming unbalanced and difficult to control [18]. When decelerating, the weight of the vehicle transfers to the front axle, increasing the friction between the front tires and the road surface [36], maximising grip at the front of the car for better turn in. If reducing trail braking and producing a quicker onset and release of the brakes (in the case of the Hall sensor), the weight of the car may shift to the rear of the vehicle earlier, hindering the vehicle’s and driver’s ability to utilise all the available grip, inducing understeer [37]. Overall, the longer TBDs that are achievable with the load cell brake sensor may contribute to a slight performance advantage when using the load cell brake.

As we strived to mimic load cell behaviour on the Hall sensor brake and created mechanically identical brake conditions, we were also interested in whether racers would be able to subjectively detect differences between the brake sensors. Interestingly, over half of participants (9/17) responded that they detected no difference in how the brakes responded in-game, while 7 out of 17 failed to correctly identify which brake was the load cell, although a slight trend towards participants reporting that the two brakes felt different when pressed was present. Given that the brakes were mechanically identical, and brake condition order was randomised across participants, we looked to determine whether differences in in-game responses may explain the performance and braking behaviour differences we were observing in our data.

**Figure 9:**
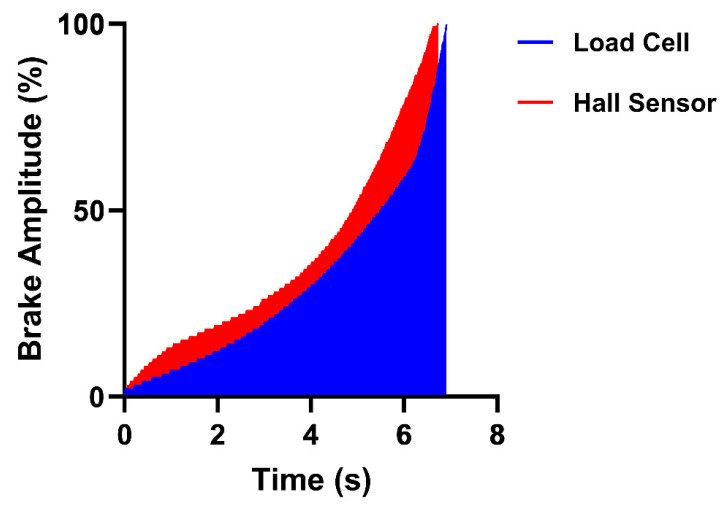
In-game response curve for the load cell brake (blue) and Hall sensor brake (red).

Subsequent analysis demonstrated that the performance difference we observed between the brake sensors may be due to the way in which they responded differently in-game and not the functioning of the sensors themselves. While we were able to demonstrate identical force–travel curves for the two sensors using the Zwick machine (as can be seen in Figure 2), we hypothesised that the conversion of the force–travel values by the game to the output of brake amplitudes values may differ. To test this, we again used a uniaxial force–displacement machine (ZwickRoell Ltd.; Worcester, UK) to press the brake pedals at a constant velocity until the brakes were fully depressed, producing consistent force–travel curves. However, this time in-game telemetry data were concurrently recorded to gather brake amplitude values to produce in-game response curves (Figure 9).

As observed in Figure 9, the in-game response curves of the braking mechanisms do differ slightly. We carried out area underneath the curve (AUC) calculations to compare the response curves of both brakes, with the Hall sensor response curve displaying a larger area (247.8% × s) than the load cell response curve (206.8% × s). The Hall sensor’s in-game response curve matches its force–travel curve, confirming we successfully mapped the load cell force–travel curve to the sensor. However, our assumption that the load cell brake’s force–travel curve would be reproduced in-game was found not to be the case.

As can be seen on the Hall sensor brake curve, peak brake amplitude is reached slightly earlier in the game compared with the load cell sensor brake (despite the actual velocity and travel of the two brake sensors being identically controlled via the Zwick machine). This may explain the difference we see in TPB values where participants were able to reach peak braking quicker with the Hall sensor brake. Furthermore, these response curves may have an impact in the slower trail braking phase (see initial phase of the response curves in Figure 9) as the Hall sensor brake amplitudes rapidly change in-game at lower amplitudes, which would make controlling trail braking using the Hall sensor difficult. As a result, it is possible that the observed performance and braking behaviour differences between the two brake sensors may be primarily influenced by the way brake force increases over time within the game (i.e., how they respond in-game).

Although we are the first to robustly assess the differences in sim racing performance between the load cell and Hall sensor brakes, there is a notable limitation within this study. As outlined extensively, although both brakes were mechanically identical (as seen in Figure 2), the in-game response of the brakes differed. Therefore, it may be the case that the difference seen in lap time and braking behaviour are due to these differences in in-game response rather than the sensors themselves. A future study may look to remap the in-game response curve associated with the load cell brake sensor to match the Hall sensor in-game response curve to better understand how braking behaviour and racing performance are impacted between the two sensors.

Our research may also have implications for broader driving simulation contexts, as the automotive industry is now transitioning to the use of brake-by-wire systems [38] and are already using Hall sensors in electronic accelerator pedals [22]. These systems may be able to utilise sensors such as the load cell and Hall sensors, with driving simulators providing the perfect testbed to evaluate their performance.

## 5. Conclusions

This study is the first to comparatively analyse the performance of two of the most common sensors used in sim racing pedals amongst sim racers. Although the load cell brake outperforms the Hall sensor brake in both lap time and braking behaviour, it is important to consider how the braking mechanisms respond in-game, despite the mechanical setup being identical, which may have contributed to the differences seen in performance, specifically contributing to the differences in the time taken to reach peak braking and the trail braking phase.

## Figures and Tables

**Table 1:** Questions participants responded throughout the testing session to ascertain perceptual differences between the brake sensor conditions.

Question	Response Options/Scale
1. How well do you think you performed with this brake (asked after each condition)?	1–7 (1: very poorly; 7: very well)
2. Was there any difference in feel between the two brakes?	Yes/No (give reasons why)
3. Was there any difference in how the two brakes responded in-game?	Yes/No (give reasons why)
4. Which brake do you think you performed better with?	Load cell/Hall sensor
5. Which brake do you think was the load cell brake?	1st condition/2nd condition

**Table 2:** Formulae for braking behaviour variables examined in this study.

Variable	Formula
Average Brake Amplitude (%)	$\frac{\sum_{1}^{n}Average\;Brake\;Amplitude}{n}$
Trail Braking Duration (s)	$\frac{\sum_{1}^{n}\left(Braking\;end\;\left(s\right)-Peak\;brake\;end\;\left(s\right)\right)}{n}$
Time to Peak Brake (s)	$\frac{\sum_{1}^{n}\left(Peak\;brake\;start\;\left(s\right)-Brake\;start\;\left(s\right)\right)}{n}$
Braking Duration (s)	$\frac{\sum_{1}^{n}\left(Brake\;end\;\left(s\right)-Brake\;start\;\left(s\right)\right)}{n}$

**Table 3:** Summary of lap time performance, braking behaviours, and braking behaviour consistency following paired samples *t*-tests. Data are expressed as means and *SD*, with significant *p* values seen in bold.

Variable	Load Cell (Mean ± SD)	Hall Sensor (Mean ± SD)	*t*-Value	*p*-Value	Effect Size (*d*)	Observed Power (1 − β)
Avg Lap Time	89.67 ± 1.63	89.97 ± 1.73	−1.932	0.071	0.469	0.443
Fastest Lap Time	88.97 ± 1.59	89.41 ± 1.53	−2.099	0.052	0.509	0.505
BA	56.27 ± 11.50	62.99 ± 13.36	−4.957	**<0.001**	1.202	0.996
TBD	1.26 ± 0.40	0.94 ± 0.34	9.891	**<0.001**	2.399	1.000
TBD CV	8.86 ± 4.08	12.41 ± 4.00	−1.426	0.213	0.582	0.214
TPB	0.38 ± 0.18	0.31 ± 0.15	3.396	**<0.004**	0.824	0.890
TPB CV	8.54 ± 4.06	15.91 ± 6.74	−1.794	0.133	0.732	0.058
BD	2.13 ± 0.27	1.92 ± 0.21	6.330	**<0.001**	1.535	0.999
BD CV	5.87 ± 1.43	6.33 ± 3.24	−0.286	0.786	0.117	0.056

**Table 4:** Summary of lap time consistency and braking amplitude consistency following Wilcoxon signed-rank tests. Data are expressed as medians and interquartile ranges.

Variable	Load Cell (Median, IQR)	Hall Sensor (Median, IQR)	*Z*-Value	*p*-Value	Effect Size (*r*)	Observed Power (1 − β)
Lap Time CV	0.55, 0.43–0.76	0.61, 0.40–1.22	−0.105	0.92	0.030	0.051
BA CV	6.33, 4.66–9.44	7.42, 4.53–9.40	−0.314	0.75	0.089	0.055

**Table 5:** Summary of responses for perception of feel, in-game response, and load cell identification following chi-square goodness-of-fit tests. Data are expressed as counts (# denotes number).

**Variable**	**# Responded Yes**	**# Responded No**	***X*** **^2^** **-Value**	***p*** **-Value**
Was there any difference in feel between the two brakes?	12	5	2.882	0.09
Was there any difference in how the two brakes responded in-game?	8	9	0.059	0.81
**Variable**	**# Correctly Identified**	**# Incorrectly Identified**	***X*** **^2^** **-Value**	***p*** **-Value**
Which brake do you think was the load cell brake?	10	7	0.529	0.47

## Data Availability

The raw data supporting the conclusions of this article will be made available by the authors on request to A.J.T.

## Supplementary Materials

The following supporting information can be downloaded at: https://www.mdpi.com/article/10.3390/s25133872/s1. File S1: Questionnaire responses. Figure S1. Utilisation of a Hall sensor in a sim racing pedal. Figure S2. Visual representation of the Hall-effect. Figure S3. Utilisation of a load cell sensor in a sim racing brake. Figure S4. Conversion of the clutch pedal to brake pedal. Figure S5. Visual representation of the protocol used in the study.

## References

[1] Sturm D., Rogers R. (2019). Not Your Average Sunday Driver: The Formula 1 Esports World Championship. Understanding Esports: An Introduction to the Global Phenomenon.

[2] Coleman M. (2025). Inside Max Verstappen’s Long-Term Goal to Make F1 Drivers out of Sim Racers. The New York Times.

[3] Opong R. How Popular Is Sim Racing? Is It Growing In Popularity? Flow Racers. https://flowracers.com/blog/how-popular-is-sim-racing/.

[4] Steam Charts Steam Charts—An Ongoing Analysis of Steam’s Concurrent Players. https://steamcharts.com/search/?q=F1.

[5] MMO Stats iRacing. Mmostats.com. https://mmostats.com/game/iracing.

[6] Jackson M.L., Croft R.J., Kennedy G.A., Owens K., Howard M.E. (2013). Cognitive Components of Simulated Driving Performance: Sleep Loss Effects and Predictors. Accid. Anal. Prev..

[7] Howard J., Bowden V.K., Visser T. (2023). Do Action Video Games Make Safer Drivers? The Effects of Video Game Experience on Simulated Driving Performance. Transp. Res. Part F Traffic Psychol. Behav..

[8] Lobjois R., Faure V., Désiré L., Benguigui N. (2021). Behavioral and Workload Measures in Real and Simulated Driving: Do They Tell Us the Same Thing about the Validity of Driving Simulation?. Saf. Sci..

[9] Lefebvre F., Malinen V., Karhulahti V. (2024). Sociohistorical Development of Sim Racing in European and Asia-Pacific Esports: A Cross-Cultural Qualitative Study. Convergence.

[10] Lappi O. (2018). The Racer’s Mind—How Core Perceptual-Cognitive Expertise Is Reflected in Deliberate Practice Procedures in Professional Motorsport. Front. Psychol..

[11] Joyce J.M., Campbell M.J., Hojaji F., Toth A.J. (2024). Less Is More: Higher-Skilled Sim Racers Allocate Significantly Less Attention to the Track Relative to the Display Features than Lower-Skilled Sim Racers. Vision.

[12] van Leeuwen P.M., de Groot S., Happee R., de Winter J.C.F. (2017). Differences between Racing and Non-Racing Drivers: A Simulator Study Using Eye-Tracking. PLoS ONE.

[13] de Frutos S.H., Castro M. (2021). Assessing Sim Racing Software for Low-Cost Driving Simulator to Road Geometric Research. Transp. Res, Procedia.

[14] Sultana M., Gheorghe L., Perdikis S. (2025). EEG Correlates of Acquiring Race Driving Skills. J. Neural Eng..

[15] Fazilat H., Toth A.J., Joyce J.M., Campbell M.J. (2024). AI-Enabled Prediction of Sim Racing Performance Using Telemetry Data. Comput. Hum. Behav. Rep..

[16] Smithies T.D., Campbell M.J., Ramsbottom N., Toth A.J. (2021). A Random Forest Approach to Identify Metrics That Best Predict Match Outcome and Player Ranking in the Esport Rocket League. Sci. Rep..

[17] Remonda A., Veas E., Luzhnica G. (2021). Comparing Driving Behavior of Humans and Autonomous Driving in a Professional Racing Simulator. PLoS ONE.

[18] Mansell S. How to Trail Brake—What Is Trail Braking and Why It’s Fast. Driver61. https://driver61.com/uni/trail-braking/.

[19] Mansell S. The Ultimate Guide to Braking. Driver61. https://driver61.com/uni/braking/.

[20] Crescentini M., Syeda S.F., Gibiino G.P. (2021). Hall-Effect Current Sensors: Principles of Operation and Implementation Techniques. IEEE Sens. J..

[21] Ramsden E. (2006). Hall-Effect Sensors: Theory and Application.

[22] Holenko K., Dykha A., Posonskiy S. The Influence of the Hall Effect on the Performance of Electronic Accelerator Pedals. Proceedings of the 2022 IEEE 4th International Conference on Modern Electrical and Energy System (MEES).

[23] Jing H., Lin Q., Liu M., Liu H. (2024). Electromechanical Braking Systems and Control Technology: A Survey and Practice. Proc. Inst. Mech. Eng. Part D J. Automob. Eng..

[24] Muller I., de Brito R., Pereira C., Brusamarello V. (2010). Load Cells in Force Sensing Analysis—Theory and a Novel Application. IEEE Instrum. Meas. Mag..

[25] Mansell S. Pedal Settings in F1 2021. Driver61. https://driver61.com/sim-racing/pedal-settings-in-f1-2021-how-to-set-your-pedals-the-right-way/.

[26] Farnell 100 kg Load Cells | Farnell® UK. Farnell.com. https://uk.farnell.com/c/sensors-transducers/sensors/force-strain-measurement/load-cells?load-capacity=100kg.

[27] Farnell Hall Effect Sensors | Farnell® UK. Farnell.com. https://uk.farnell.com/c/sensors-transducers/sensors/magnetic-sensors/hall-effect-sensors.

[28] Hopkins W.G. (2000). Measures of Reliability in Sports Medicine and Science. Sports Med..

[29] Lovie P., Everitt B.S., Howell D.C. (2005). Coefficient of Variation. Encyclopedia of Statistics in Behavioral Science.

[30] RacingMat, DXTweakX, Ver. 2020-08-06. https://www.xsimulator.net/community/marketplace/dxtweak2-change-calibration-and-deadzone-settings-of-a-directinput-game-controller.245/.

[31] (2018). Metallic Materials—Calibration and Verification of Static Uniaxial Testing Machines—Part 1: Tension/Compression Testing Machines—Calibration of the Force-Measuring System.

[32] Logitech, G HUB, Ver. 2024.3.553733. https://www.logitechg.com/en-gb/innovation/g-hub.html.

[33] Shechtman O., Doi S.A.R., Williams G.M. (2013). The Coefficient of Variation as an Index of Measurement Reliability. Methods of Clinical Epidemiology.

[34] Parra A., Tavernini D., Gruber P., Sorniotti A., Zubizarreta A., Pérez J. (2021). On Pre-Emptive Vehicle Stability Control. Veh. Syst. Dyn..

[35] Zarkadis K., Velenis E., Siampis E., Longo S. Predictive Torque Vectoring Control with Active Trail-Braking. Proceedings of the 2018 European Control Conference (ECC).

[36] Velenis E., Tsiotras P., Lu J. (2008). Optimality Properties and Driver Input Parameterization for Trail-Braking Cornering. Eur. J. Control.

[37] Driver61 How to Trail Brake: A Step-by-Step Guide. YouTube. https://www.youtube.com/watch?v=tvcuGoVhpxw.

[38] Hua X., Zeng J., Li H., Huang J., Luo M., Feng X., Xiong H., Wu W. (2023). A Review of Automobile Brake-By-Wire Control Technology. Processes.

