# Supplementary material for: A Comparative Performance Analysis of Load Cell and Hall-Effect Brake Sensors in Sim Racing
Source: Sensors (Basel). 2025 Jun 21;25(13):3872. doi: 10.3390/s25133872 (PMC12251626; doi:10.3390/s25133872)
Supplement: Supplementary file 1 [file sensors-25-03872-s001.zip › Supplementary Figure Captions_R1.pdf]

Supplementary Figure Captions

Figure S1 (Utilisation of the Hall sensor in a sim racing pedal)

Example of a Hall sensor mechanism for the throttle of the Logitech G Pro pedal set (Logitech; CU). (**a**) Magnet on the pedal that creates the Hall effect when interacting with the sensor board, (**b**) Hall sensor board.

Figure S2 (Visual representation of the Hall-effect)

Diagram illustrating the Hall effect from Ramsden (2006). **(a)** *I* current is being carried through a conductive material. **(b)** A magnetic field is applied perpendicular to the flow of current, two probes on opposite sides along the plate can detect a voltage change.

Figure S3 (Utilisation of a load cell sensor in a sim racing brake)

Load cell sensor placed underneath the brake pedal from the Logitech G Pro pedal set.

Figure S4 (Conversion of the clutch pedal to brake pedal)

The spring mechanism from the clutch pedal on the left was replaced with the load cell piston on the right.

Figure S5 (Protocol visual)

A visualisation of the protocol carried out in the current study.
