# Supplementary figures and images for: A Comparative Performance Analysis of Load Cell and Hall-Effect Brake Sensors in Sim Racing
Source: Sensors (Basel). 2025 Jun 21;25(13):3872. doi: 10.3390/s25133872 (PMC12251626; doi:10.3390/s25133872)

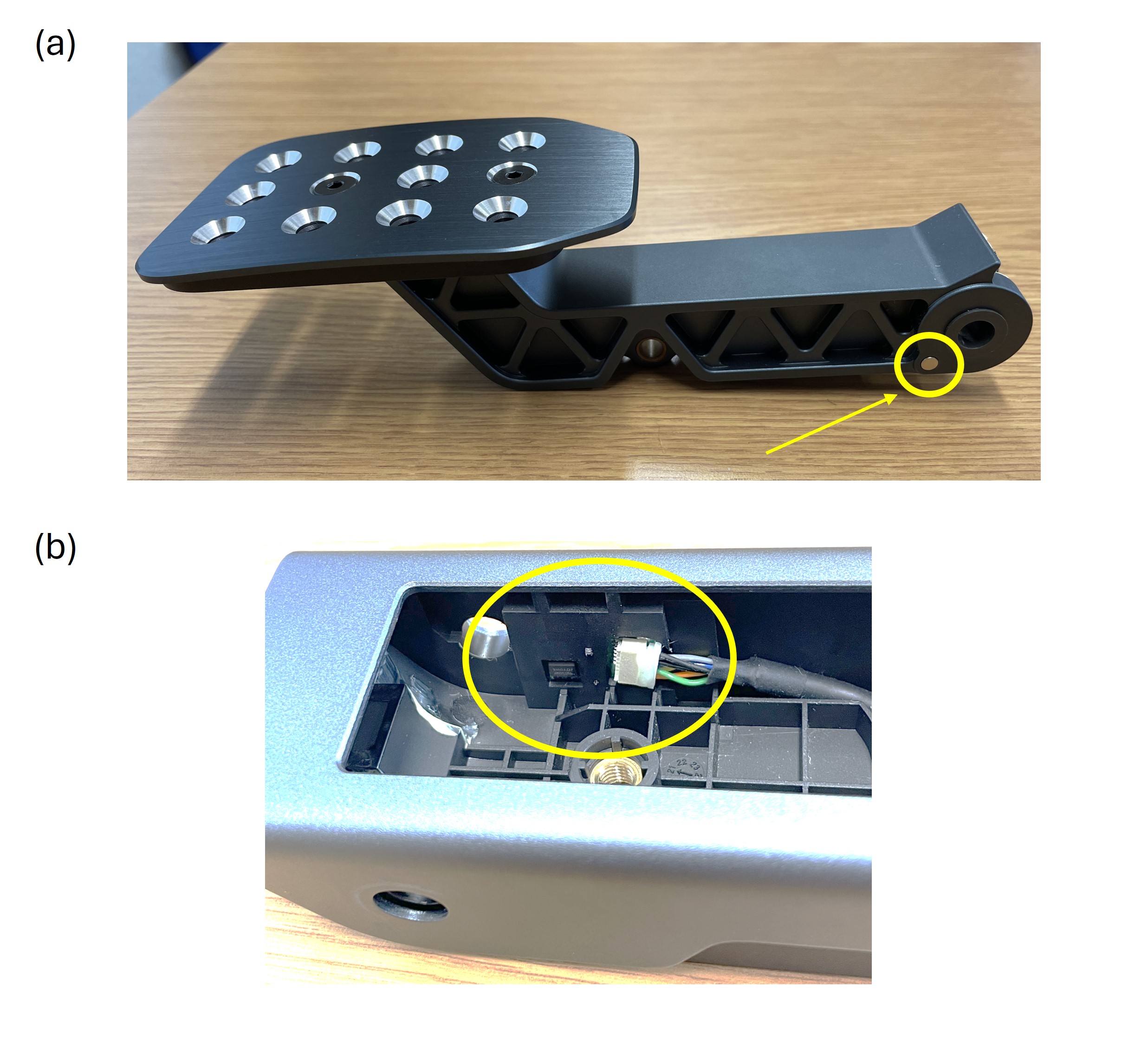

Supplement: Supplementary file 1 [file sensors-25-03872-s001.zip › Supplementary Figure S1_R1.jpg]

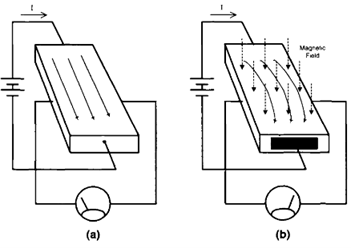

Supplement: Supplementary file 1 [file sensors-25-03872-s001.zip › Supplementary Figure S2_R1.tif]

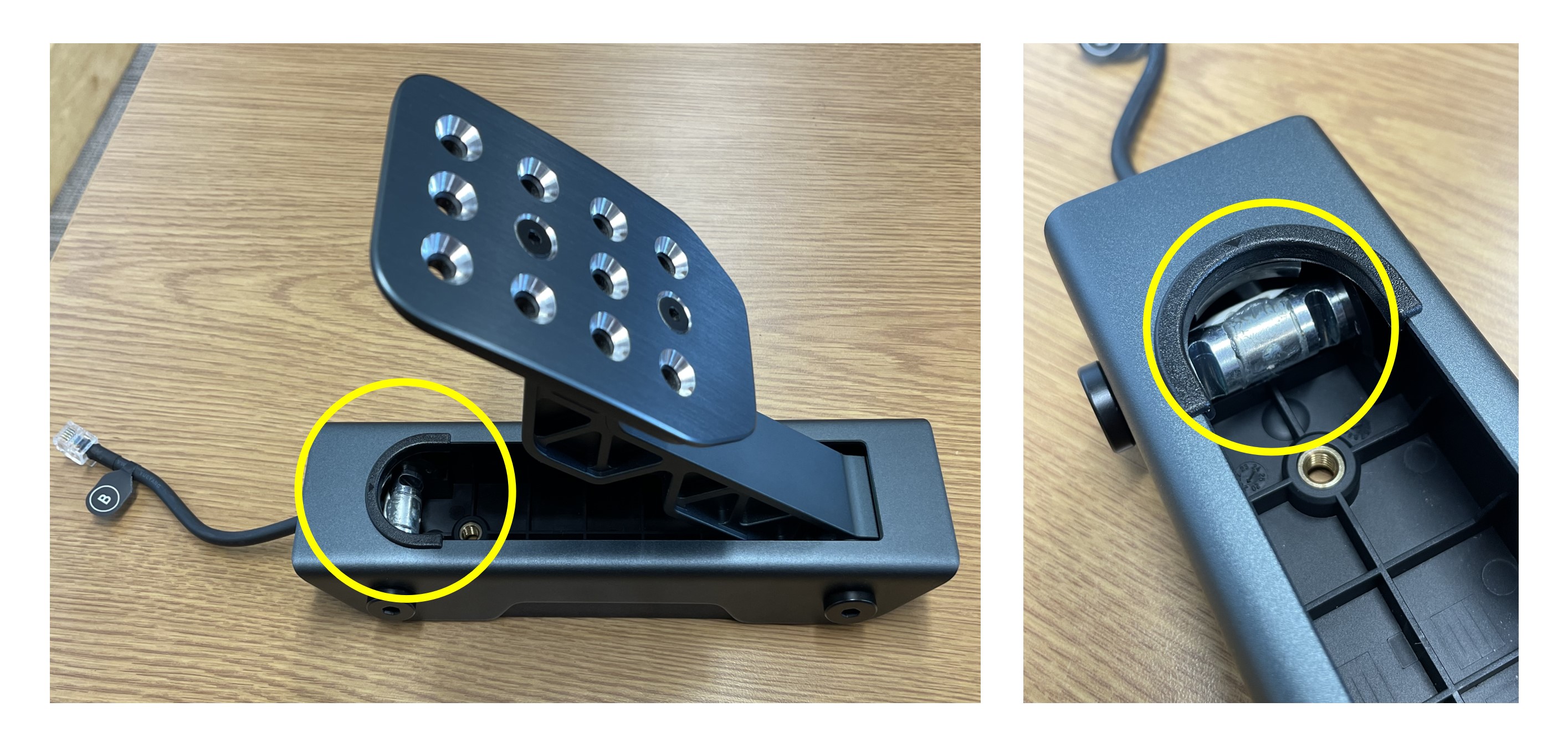

Supplement: Supplementary file 1 [file sensors-25-03872-s001.zip › Supplementary Figure S3_R1.jpg]

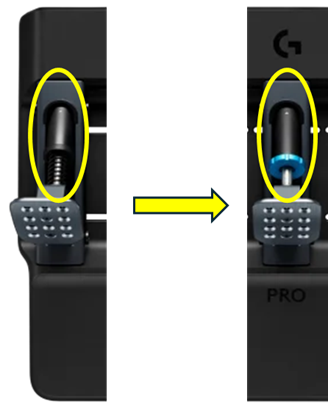

Supplement: Supplementary file 1 [file sensors-25-03872-s001.zip › Supplementary Figure S4_R1.tif]

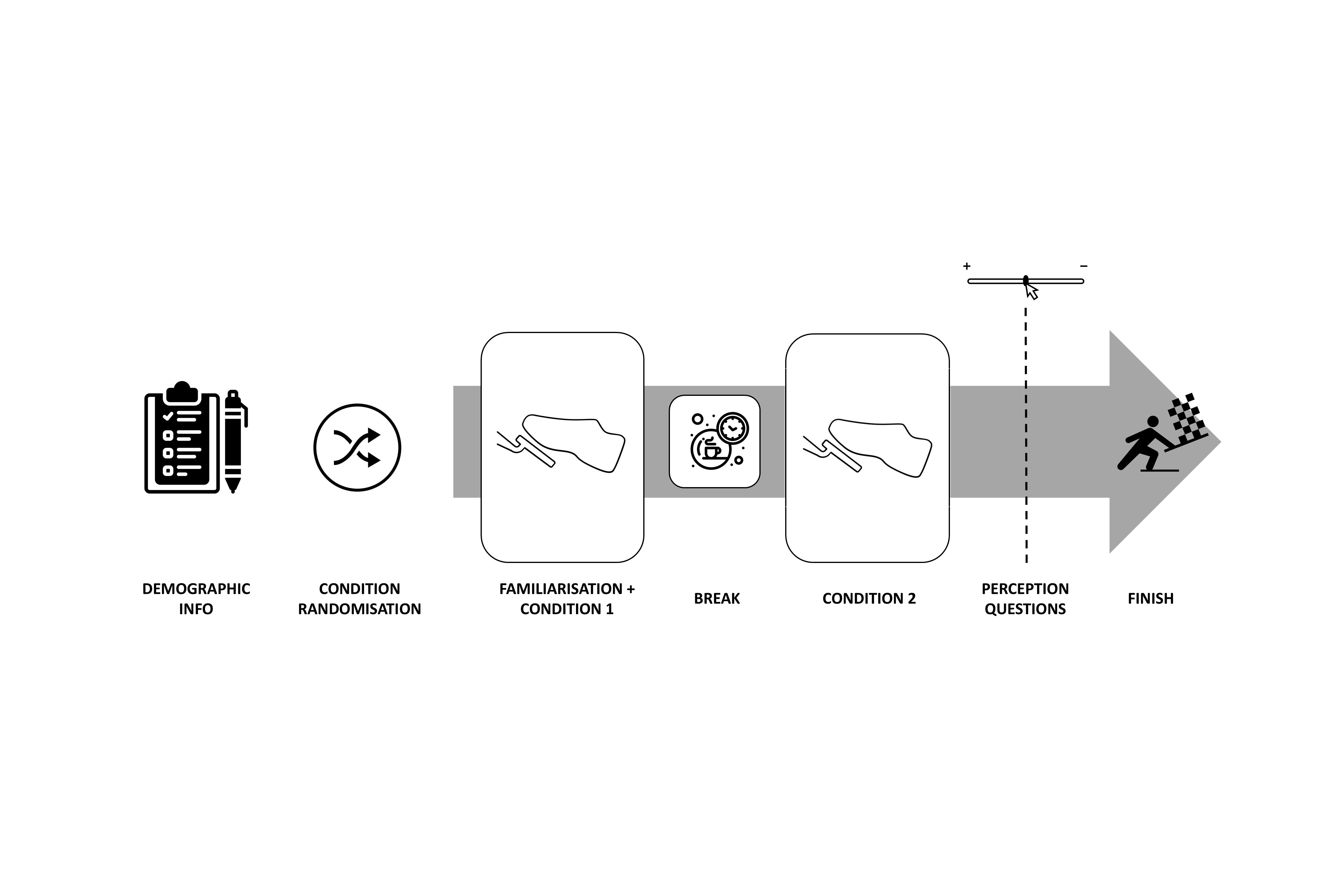

Supplement: Supplementary file 1 [file sensors-25-03872-s001.zip › Supplementary Figure S5_R1.tif]
